# Effects of combined extreme cold and drought stress on growth, photosynthesis, and physiological characteristics of cool-season grasses

**DOI:** 10.1038/s41598-023-49531-1

**Published:** 2024-01-02

**Authors:** Juanxia Li, Xiaoming Bai, Fu Ran, Caizhong Zhang, Yubang Yan, Ping Li, Hui Chen

**Affiliations:** 1 College of Grassland Science, Gansu Agricultural University, Lanzhou, 730070 China; 2 Key Laboratory of Grassland Ecosystem of the Chinese Ministry of Education, Lanzhou, 730070 China

**Keywords:** Ecology, Plant sciences, Environmental sciences

## Abstract

Abiotic stress is an important factor affecting turf establishment and limiting the sustainability of the turf industry. To alleviate the effects of combined cold and drought stress in cold- and drought-prone regions, it is essential to select and introduce turfgrass germplasms that are suitable for these conditions for successful turf establishment. Thus, we evaluated the effects of combined extreme cold and drought stress on the morphological, plant leaf functional, photosynthetic, and physiological and biochemical traits of 16 wild annual bluegrass (*Poa annua*) germplasms. We found that there were significant differences (*P* < 0.05) among different provenances, combined cold and drought stress, and the main interaction factors. Combined cold and drought stress altered the morphological characteristics of the 16 germplasms to varying degrees. Furthermore, combined cold and drought stress significantly reduced the net photosynthetic rate (Pn), stomatal conductance (gs), transpiration rate (Tr), instantaneous water use efficiency (WUE), chlorophyll content, chlorophyll fluorescence parameters, accumulated intercellular CO_2_ concentration (Ci), and relative electrical conductivity (REC) and malondialdehyde (MDA), proline (Pro), soluble protein (SP), soluble sugar (SS), superoxide anion (O_2_^.-^), hydrogen peroxide (H_2_O_2_), and hydroxyl radical (·OH) and other active oxygen, and increased the superoxide dismutase activity (SOD), peroxidase activity (POD), catalase activity (CAT), ascorbate peroxidase activity (APX) and glutathione reductase (GR) activities. Comprehensive evaluation using principal component analysis (PCA), membership function analysis, and clustered heatmaps indicated that the ‘HZ’ germplasm had stronger combined cold and drought tolerance, whereas the ‘ZQ’ germplasm was more sensitive to combined cold and drought, which was roughly consistent with the order of morphological damage symptoms. Therefore, it is recommended to use the ‘HZ’ germplasm for planting projects in cold- and drought-prone areas, while the ‘ZQ’ germplasm is more suitable for use under warmer and non-water-deficient conditions.

## Introduction

Abiotic stresses, such as extreme temperature, drought, salinity, and oxidative stress pose serious threats to agriculture and lead to environmental degradation. Abiotic stresses result in a series of morphological, physiological, biochemical, and molecular changes that negatively affect plant growth and productivity^1^. It is believed that cold and drought are the major factors limiting plant growth and production among all abiotic stressors globally^2^. Due to rapid and dynamic changes in the global environment, plants are increasingly and frequently exposed to a combination of various abiotic stresses^3^, such as combined cold and drought, which can cause more severe damage to plants than an individual stressor and are not conducive to agricultural production and environmental sustainability^4^. Moreover, the responses of plant to combined stresses are unique and cannot be inferred directly from the single-stress responses^5,6^. Most studies involving cold and drought stress have focused on the response of plants to individual stress, and fewer studies on combined stress^4,7^. The combined effect of cold and drought stress can induce significant changes in plant morphology, such as leaf wilting and senescence, stem and root growth inhibition, and fruit damage, severely reducing crop productivity^8,9^. Plants can develop a variety of defense mechanisms to counteract the damage caused by stress^10^. Generally, cold (usually below 10 °C) and drought stress can affect photosynthesis and nutrient uptake in plants^11,12^. Both cold and drought stress normally lead to a disruption in chloroplast ultrastructure, hinder the electron transfer of PSII in chloroplast membranes, and reduce chloroplast pigment synthase activity, resulting in insufficient raw material supply for chlorophyll synthesis, thus slowing plant metabolism and reducing chlorophyll content in leaves^4^. The response patterns of plants to cold or drought stress individually are similar^5^, but the effects of combined cold and drought stress can be more pronounced than those of each stress applied individually.

The combination of cold and drought stress can result in the accumulation of large amounts of reactive oxygen species (ROS), such as superoxide anion (O_2_^·-^), hydrogen peroxide (H_2_O_2_), and hydroxyl radicals (·OH) and other active oxygen, impairing plant function by causing protein denaturation, lipid peroxidation, DNA damage, and enzyme inhibition^6^. Additionally, numerous studies have shown that the mitigation of oxidative damage induced by environmental stress and the enhancement of plant resistance may be related to the activation of antioxidant defense systems^13,14^. Plants will activate the antioxidant enzyme system and nonenzymatic system mechanisms to ensure that reactive oxygen species protection is provided under stress, thereby maintaining normal plant growth and metabolism. Among them, the activities of antioxidant enzymes such as superoxide dismutase (SOD), peroxidase (POD), catalase (CAT), ascorbate peroxidase (APX), and glutathione reductase (GR) can effectively regulate metabolic homeostasis in plants^15^. SOD not only acts as the first line of defense in plants for ROS scavenging but is also the principal scavenger of O_2_^·-^. The function of SOD is mainly to convert excess O_2_^·-^ into H_2_O_2_ and O_2_; H_2_O_2_ is further converted through POD into H_2_O, which is harmless to cells^16^. In the AsA-GSH cycle, APX plays a critical role in catalyzing H_2_O_2_, while GR provides substrates for APX mainly through the formation of AsA and GSH, thereby scavenging excess ROS^17^. The content of malondialdehyde (MDA) and relative electrical conductivity (REC) can indicate the degree of cellular membrane lipid peroxidation and the strength of the plant response to adverse conditions^18,19^. Proline (Pro), soluble sugar (SS) and soluble protein (SP) are essential osmoregulatory substances involved in plant response to various environmental stresses, which in turn prevent cellular dehydration^20^.

Northwest China has an arid and semi-arid climate with limited rainfall, water scarcity, cold and dry winters, and plants are subjected to long-term cold and drought stress^21,22^. Given these climatic conditions, it is a challenge to improve these ecosystems. The use of effective and systematic solutions is required to redevelop these ecosystems through the introduction of cold- and drought-resistant species to reduce the impacts of extreme temperature and water scarcity and to promote sustainable agriculture. Annual bluegrass (*Poa annua*) is one of the most widely distributed plant species in the world, with a short life history, strong reproductive capacity, resistance to trampling and pruning, strong stress resistance, and rapid growth. It serves as an excellent lawn grass and ecological restoration grass. Due to its comprehensive survival strategy, *Poa annua* plays an important role in promoting ecosystem productivity, species diversity, and ecological functions in urban green spaces^23,24^. This makes the selection and introduction of this turfgrass the perfect solution for restoring vegetation cover on ecologically degraded lands. However, a comprehensive study should be performed to evaluate the response of plant materials based on their geographical origins before they are planted to mitigate better problems related to urban landscaping, sports field turf establishment, and road berm construction in cold- and drought-prone areas. In this study, we studied the responses of *Poa annua* germplasms from 16 provenances under extreme combined cold and drought stress to determine the feasibility of introducing this species in cold- and drought-prone areas. The morphological, photosynthetic, physiological, and biochemical responses of different *Poa annua* germplasms to combined extreme cold and drought stress were determined, and the aim was to observe the effects of combined extreme cold and drought stress on different germplasms of *Poa annua* and assess the degree of tolerance of 16 *Poa annua* germplasms to combined cold and drought stress. In addition, using comparisons of the resistance of different *Poa annua* germplasms to combined temperature and water stress, we screened the germplasms that provided the best growth results for planting in urban landscaping projects in cold- and drought-prone regions of the world.

## Results

### Effects of combined cold and drought stress on plant phenotypes

**Figure 1:**
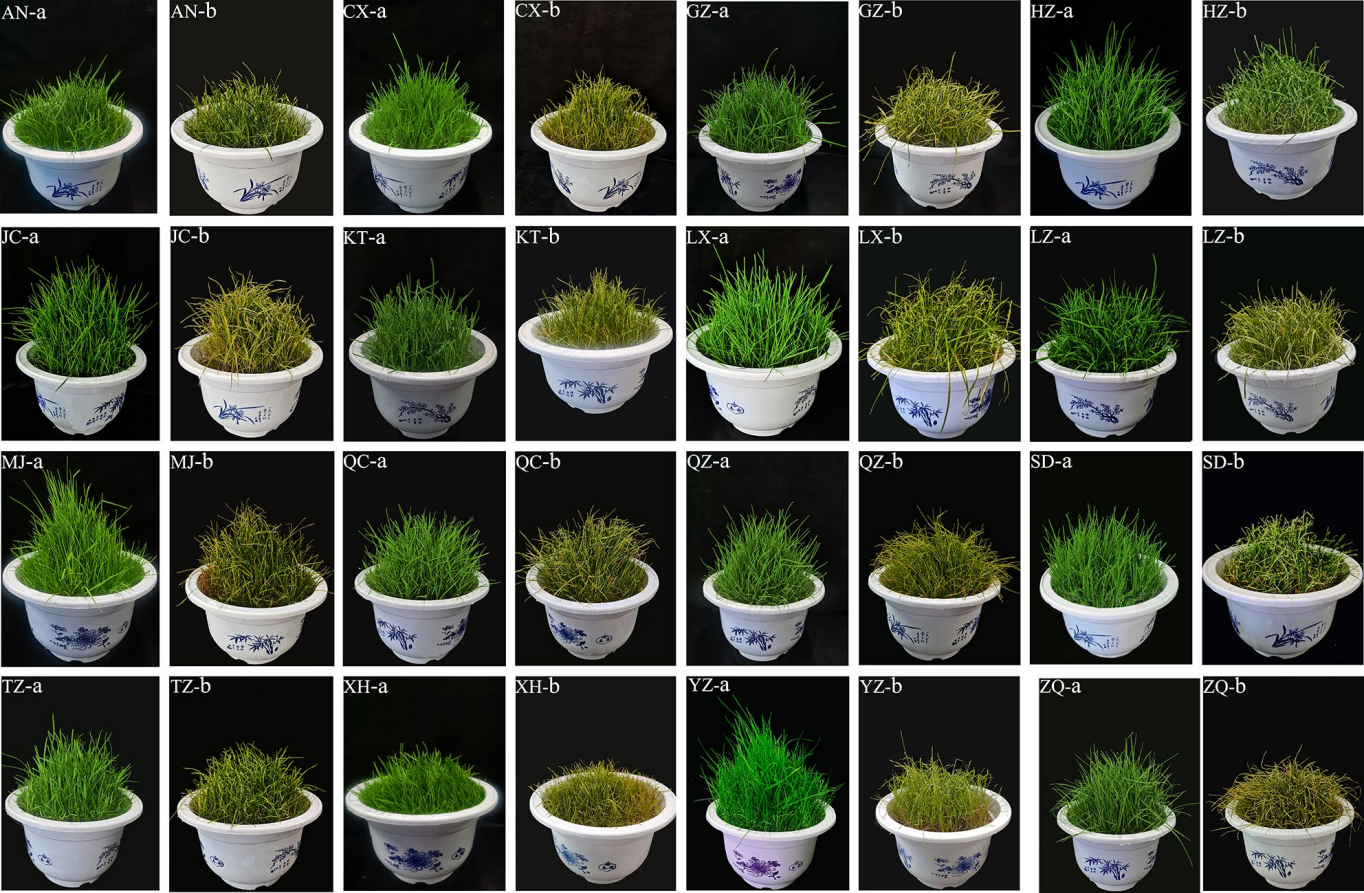
Comparison of phenological symptoms of each *Poa annua* seedling in the nonstressed and stressed groups. (**a**) nonstressed group germplasms; (**b**) stressed group germplasms.

The phenotypic symptom changes of different *Poa annua* germplasms under combined cold and drought stress were observed in this study (Fig. 1). The color of 16 germplasms was normal and the plants grew normally with regular watering (the soil moisture content was 80% of the maximum field capacity) at room temperature (25 °C). The upper leaves of 16 seedlings showed symptoms such as freezing spots, wilting, and curling after 24 h of combined cold and drought stress. Among them, more leaves of the ‘ZQ’ germplasm turned yellow, wilted, and curled, and the stalks turned black-brown. While seedlings of the ‘HZ’ germplasm had some of the upper margins of the leaves wilted and curled and showed freezing spots. Therefore, according to the phenotypic symptoms, it can be preliminarily evaluated that the resistance of the ‘HZ’ germplasm was higher, while that of the ‘ZQ’ germplasm was lower.

### Combined analysis of provenance, combined stress and provenance x combined stress

The analysis of variance (ANOVA) of the provenance and combined cold and drought stress factors showed significant differences in the leaf functional traits, photosynthetic characteristics, and physiological and biochemical traits (*P* ≤ 0.05) (Additional file 1: Table S1), which will be helpful to screen out the *Poa annua* germplasm with strong combined cold and drought resistance. Our results also indicated that there were significant differences in the interactions between the provenances and the combined cold and drought stress, showing that the resistance to the combined cold and drought stress of *Poa annua* was regulated by its genetic structure, temperature and moisture, and interactions. The genetic variation coefficients of thirty traits of 16 *Poa annua* germplasms confirmed the existence of genetic differences under combined stress; the coefficient of variation for thirty traits ranged from 8.26% (qN) to 74.97% (gs), with a mean value of 32.09%.

### Effects of combined cold and drought stress on the leaf functional traits of 16 the *Poa annua* germplasms

**Figure 2:**
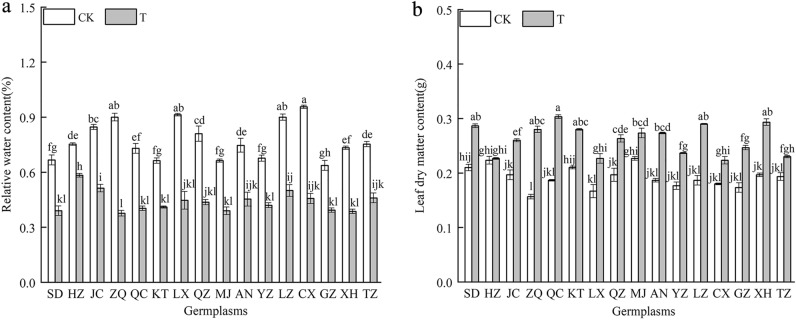
Effect of combined cold and drought stress on the leaf functional traits of 16 *Poa annua* germplasms. Values are means ± standard deviation (n = 3). Different letters indicate significant differences among treatments and germplasms (*P* < 0.05).

As shown in Fig. 2, the RWC of 16 *Poa annua* germplasms under combined cold and drought stress were significantly decreased, while the LDMC were significantly increased (*P* < 0.05). The RWC directly affects the physiological metabolism of plants. 16 germplasms under combined cold and drought stress reduced the adverse effects by decreasing intracellular water content and increasing cytosol concentration. Among them, the ‘ZQ’ germplasm showed the largest decrease in the RWC, which revealed a significant reduction of 58.15% compared with non-stressed seedlings (*P* < 0.05). The LDMC of the ‘ZQ’ germplasm showed significant differences compared to other germplasms under stress (*P* < 0.05).

### Effect of combined cold and drought stress on photosynthetic characteristics of the 16 *Poa annua* germplasms

#### Effect of combined cold and drought stress on the chlorophyll content in the 16 germplasms

**Figure 3:**
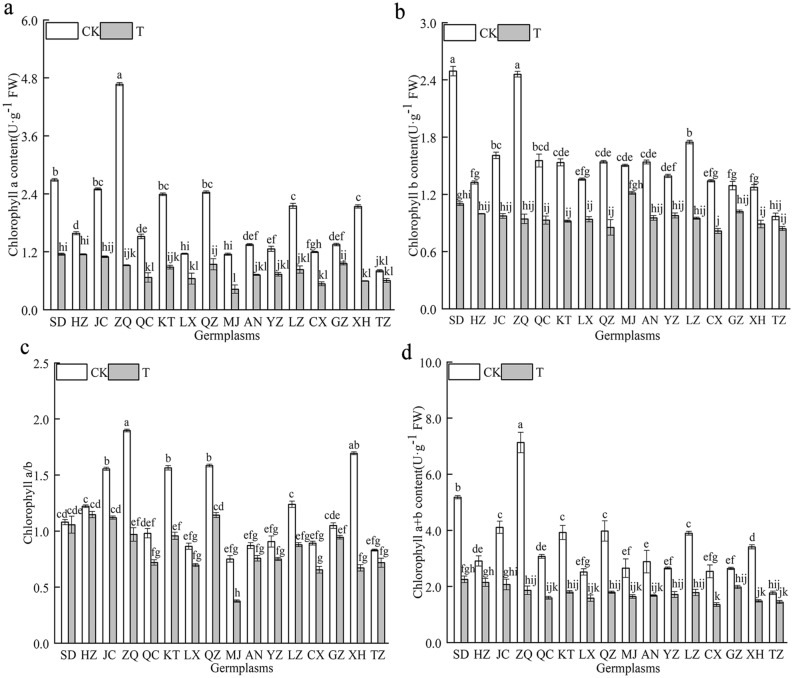
Effect of combined cold and drought stress on the chlorophyll content of different germplasms.

Chlorophyll is an essential cofactor for photosynthesis, and maintaining normal chlorophyll status and levels is critical for photosynthetic efficiency and carbon sequestration, which directly affects plant growth and development under various abiotic stresses. The Chl a, Chl b, Chl a + b contents and Chl a/b of 16 *Poa annua* germplasms were significantly decreased (*P* < 0.05) under combined cold and drought stress (Fig. 3). Among them, the ‘TZ’ germplasm showed the smallest decrease in the Chl a, Chl b and Chl a + b contents, which decreased by 24.93%, 13.23% and 18.54% compared with nonstressed germplasms, respectively. The Chl a, Chl b, and Chl a + b contents of the ‘ZQ’ germplasm showed the greatest decrease (*P* < 0.05). The Chl a/b of the ‘XH’ germplasm indicated the greatest decrease under combined cold and drought stress, which was significantly reduced by 60.39% compared with nonstressed germplasms (*P* < 0.05), demonstrating that photosynthesis was highly sensitive to stress.

#### Effect of combined cold and drought stress on the photosynthetic gas exchange parameters of the 16 germplasms

**Figure 4:**
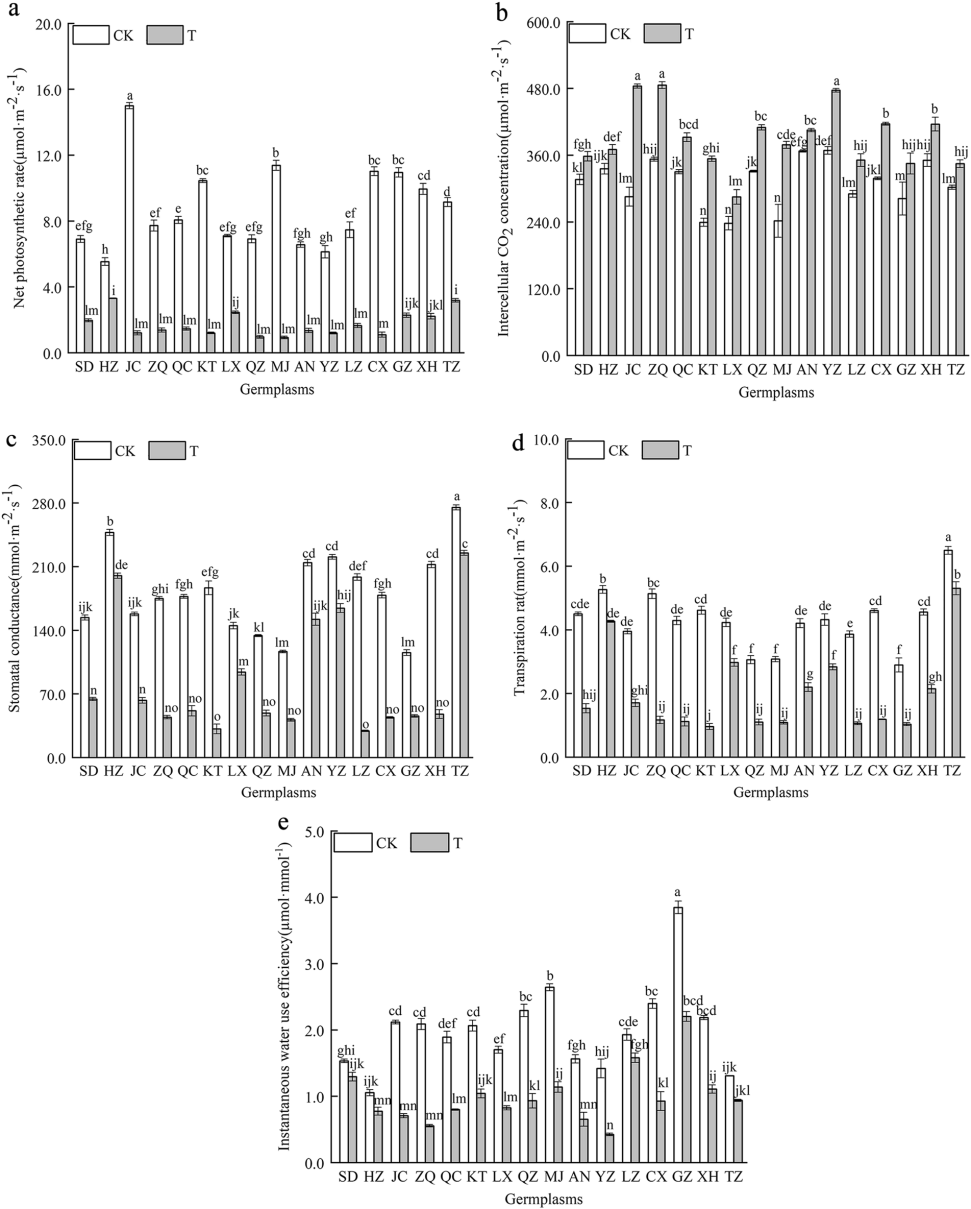
Effect of combined cold and drought stress on the photosynthetic gas exchange parameters of different germplasms.

The Pn of the ‘JC’ germplasm was significantly higher than that of other germplasms under nonstressed conditions (*P* < 0.05) (Fig. 4). The Pn, gs, Tr and WUE of 16 *Poa annua* germplasms decreased significantly under combined cold and drought stress, while Ci increased significantly (*P* < 0.05). The greatest decrease in the Pn was observed in the ‘JC’ germplasm under combined cold and drought stress, whereas the smallest decreases in the Pn, gs, Tr, and WUE were observed in the ‘HZ’ germplasm, which showed significant reductions of 40.36%, 19.24%, 18.99%, and 26.46%, respectively, compared with nonstressed germplasms (*P* < 0.05). The smallest increase in the Ci was observed in the ‘HZ’ germplasm and the largest increase was observed in the ‘JC’ germplasm under combined cold and drought stress, which significantly increased by 69.65% (*P* < 0.05) compared to nonstressed germplasms.

#### Effect of combined cold and drought stress on the chlorophyll fluorescence parameters in the 16 germplasms

**Figure 5:**
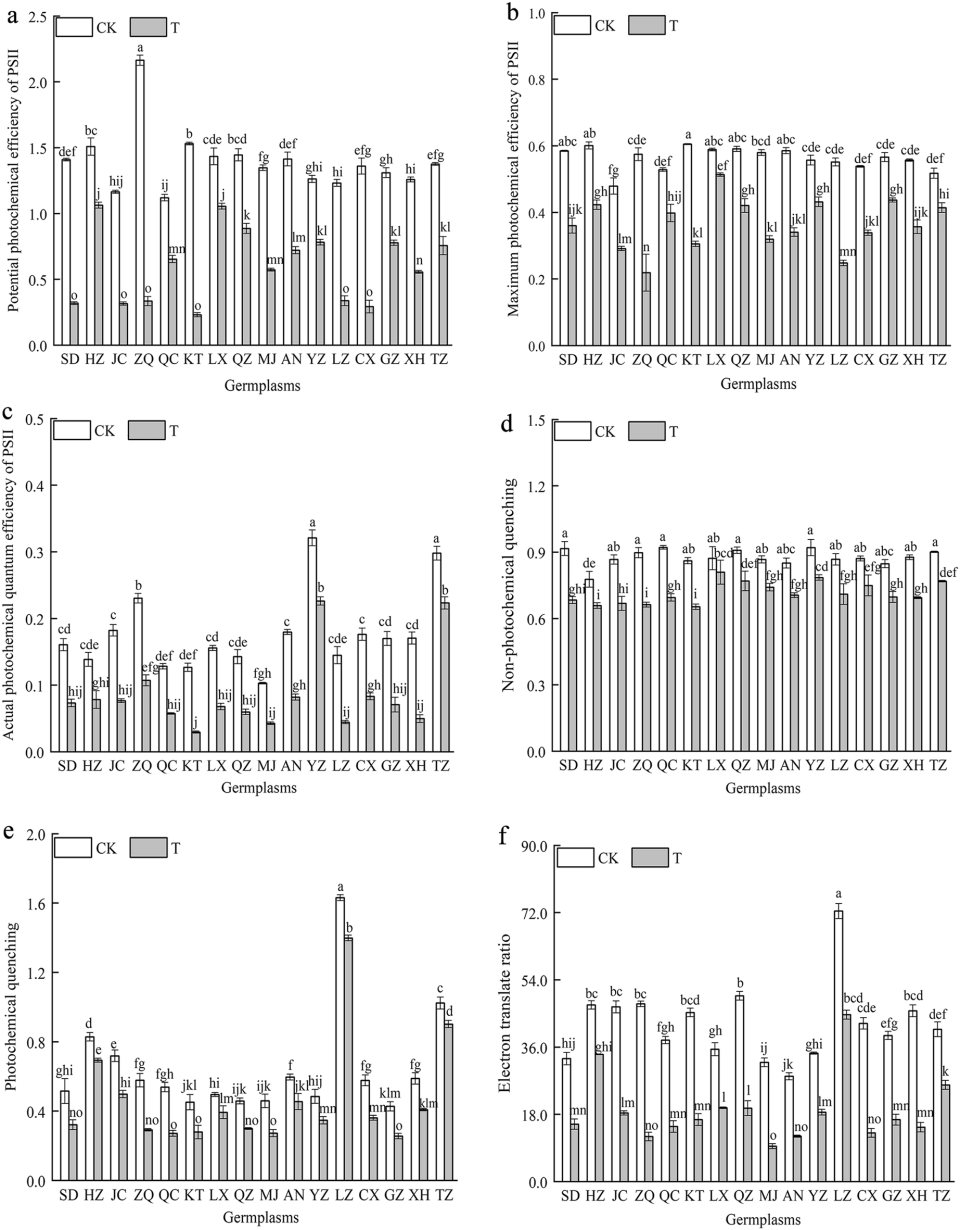
Effect of combined cold and drought stress on the chlorophyll fluorescence parameters of different germplasms.

The Fv/Fo of the ‘ZQ’ germplasm was significantly higher (*P* < 0.05) than that of other germplasms under nonstressed conditions (Fig. 5). In contrast, the Fv/Fo, Fv/Fm, ΦPSII, qN, qP and ETR of 16 *Poa annua* germplasms were reduced significantly under combined cold and drought stress (*P* < 0.05). Among them, the Fv/Fo, Fv/Fm, ΦPSII, qN, and ETR of the ‘ZQ’ germplasm decreased the most on average under stress conditions, which showed significant reductions of 84.55%, 61.92%, 53.54%, 72.60%, and 74.70%, respectively, compared with those of nonstressed germplasms (*P* < 0.05). This revealed that the effective electron transfer efficiency of PSII in the ‘ZQ’ germplasm was greatly reduced under stress conditions, and the photosynthetic system was impaired, which greatly reduced the plant’s ability to utilize light energy. In addition, the qP of the ‘HZ’ germplasm exhibited the smallest decrease under stress, whereas the ‘ZQ’ germplasm showed the largest decrease, which was sharply reduced by 30.67% compared with the nonstressed germplasms (*P* < 0.05).

### Effect of combined cold and drought stress on REC and MDA content of the 16 *Poa annua* germplasms

**Figure 6:**
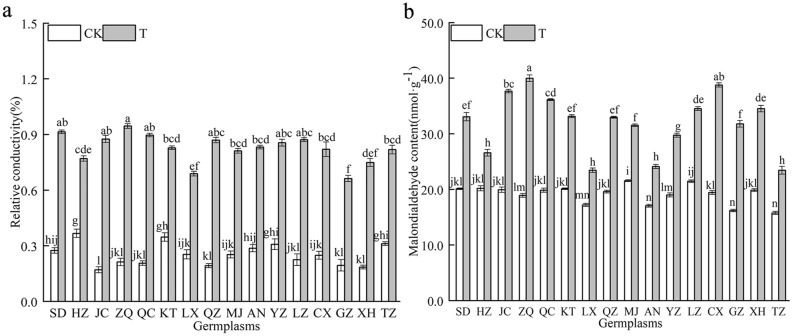
Effect of combined cold and drought stress on the REC and MDA content of different germplasms.

The REC of 16 germplasms increased significantly under the combined cold and drought stress (*P* < 0.05) (Fig. 6). The smallest increase in REC was observed in the ‘HZ’ germplasm, while the largest increase was observed in the ‘ZQ’ germplasm, which significantly increased by 345.13% compared to nonstressed germplasms (*P* < 0.05), and the REC of the ‘ZQ’ germplasm was significantly different from other germplasms. The MDA content of the 16 *Poa annua* germplasms increased rapidly under stress (*P* < 0.05), suggesting that peroxidation was enhanced with the reduction of temperature and moisture in *Poa annua* seedlings with the enhancement of membrane lipid superoxide. Among them, the ‘LX’ and ‘HZ’ germplasms showed a smaller increase in the content of MDA, which significantly increased by 36.17% and 31.67% compared to nonstressed germplasms, respectively (*P* < 0.05). The ‘ZQ’ germplasm showed the largest increase, which was outstandingly increased by 111.36% compared to the nonstressed germplasms (*P* < 0.05).

### Effect of combined cold and drought stress on the reactive oxygen species levels in 16 *Poa annua* germplasms

**Figure 7:**
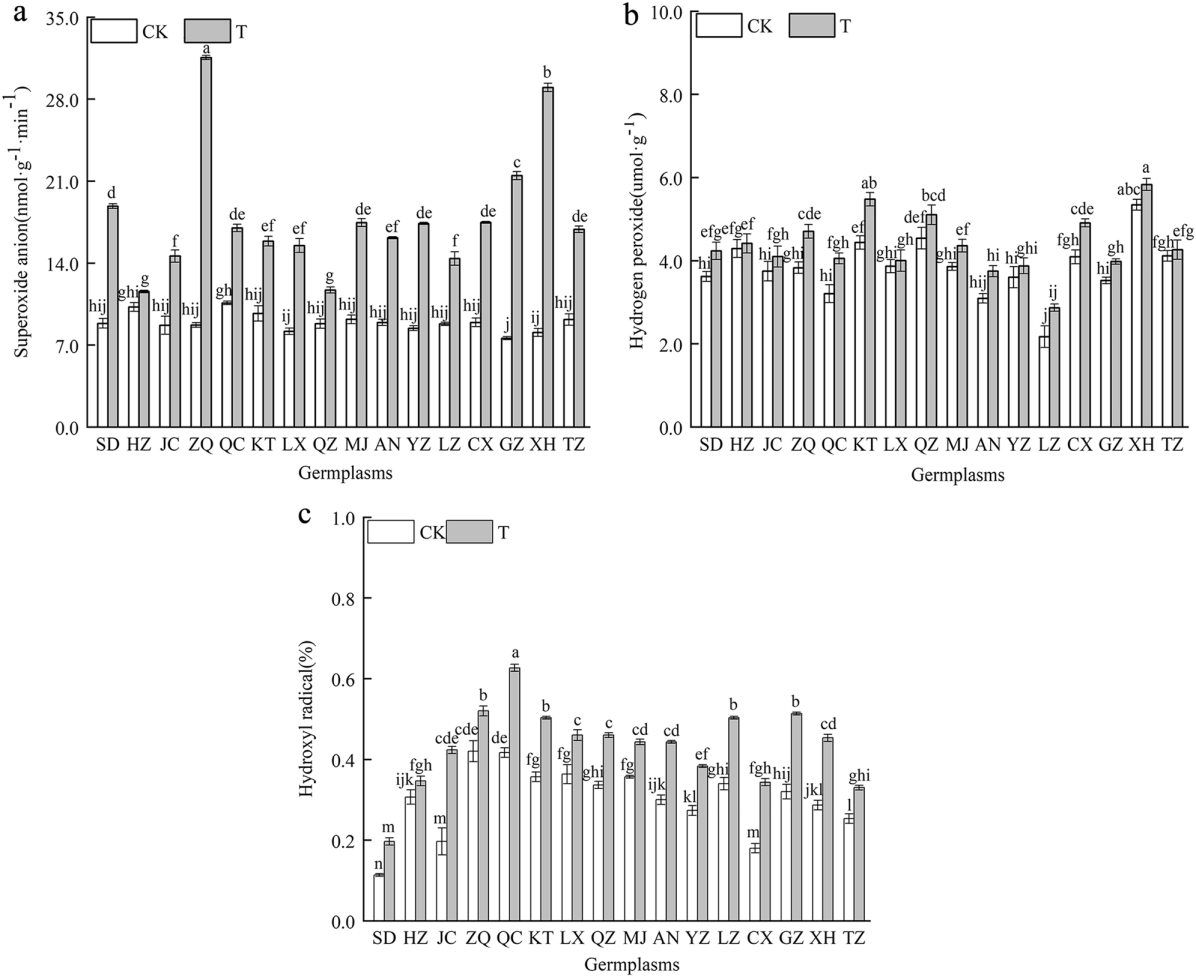
Effect of combined cold and drought stress on the reactive oxygen levels in different germplasms.

As shown in Fig. 7, O_2_^·-^ increased significantly in 16 *Poa annua* germplasms under combined cold and drought stress (*P* < 0.05). Among them, the O_2_^·-^ increase of the ‘HZ’ germplasm was the smallest, and the ‘ZQ’ germplasm was the largest. The H_2_O_2_ and ·OH of 16 *Poa annua* germplasms were increased under combined cold and drought stress. Among them, the 'HZ’ germplasm showed the smallest increase in both H_2_O_2_ and ·OH, which increased by 2.79% and 13.04% compared with nonstressed germplasms, respectively (*P* < 0.05), and these results suggested that excessive ROS production may be the main cause of leaf damage in *Poa annua*.

### Effect of combined cold and drought stress on the antioxidant enzyme activities in the 16 *Poa annua* germplasms

**Figure 8:**
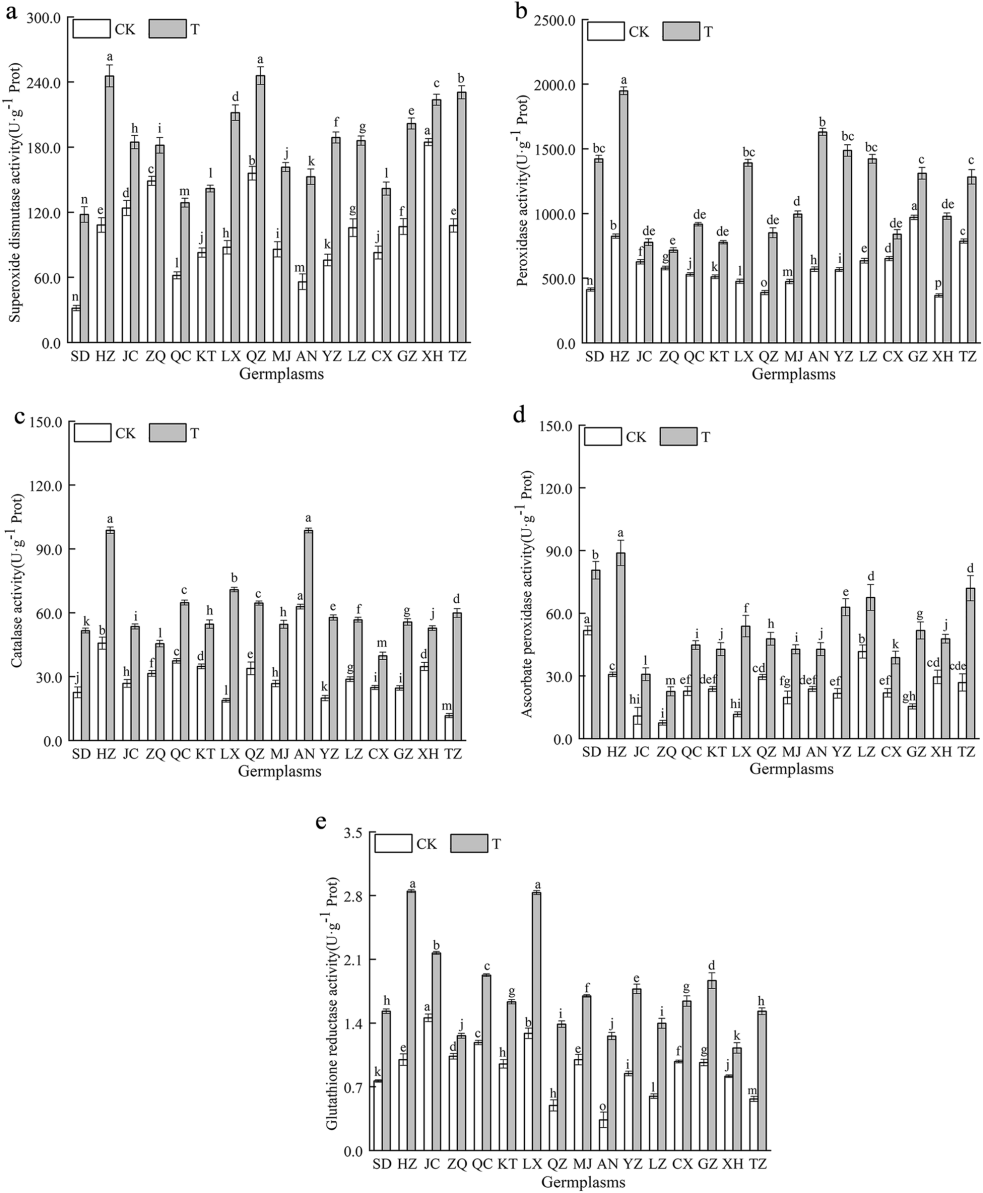
Effect of combined cold and drought stress on the antioxidant enzyme activities of different germplasms.

As shown in Fig. 8, the activities of SOD, POD, CAT, APX and GR were significantly increased in 16 *Poa annua* germplasms under combined cold and drought stress (*P* < 0.05), indicating that the enzyme activities of antioxidant systems were enhanced in all *Poa annua* under combined cold and drought stress. Among them, the activities of POD and CAT in the ‘ZQ’ germplasm showed the smallest increase, which were significantly increased by 24.19% and 44.44% compared to the nonstressed germplasms (*P* < 0.05), respectively. The CAT activity of the ‘TZ’ germplasm increased by 414.18% (*P* < 0.05), and there were significant differences between the ‘TZ’ germplasm and other germplasms. In addition, the APX activity of the ‘HZ’ germplasm increased the most, while the SOD activity of the ‘SD’ germplasm increased the most under the combined cold and drought stress, which significantly increased by 2.90 and 2.73 times, respectively, compared with the unstressed germplasms (*P* < 0.05). The greatest increase in GR activity was observed in the ‘HZ’ germplasm, while the smallest increase was observed in the ‘ZQ’ germplasm under stress (*P* < 0.05). This reflected that combined cold and drought stress enhanced the activity of antioxidant system enzymes in *Poa annua*, thereby increasing the stress tolerance of the germplasm.

### Effect of combined cold and drought stress on the osmoregulatory substances in the 16 *Poa annua* germplasms

**Figure 9:**
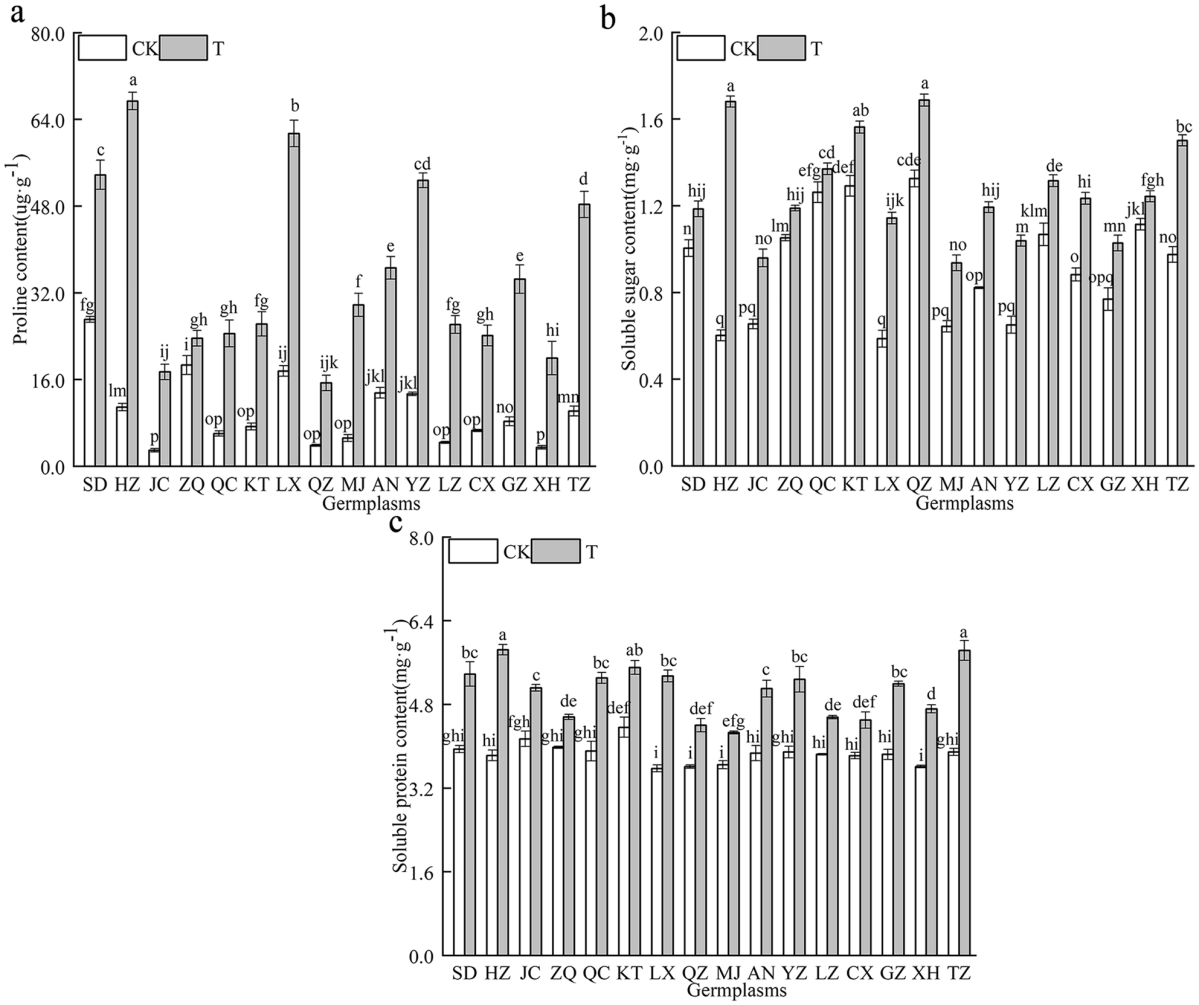
Effect of combined cold and drought stress on the osmoregulatory substances in different germplasms.

The Pro and SP contents of 16 *Poa annua* germplasms were significantly increased under combined cold and drought stress (*P* < 0.05) (Fig. 9), but the osmotic substance contents of the ‘HZ’ germplasm were the highest, increasing by 6.18 and 1.53 times compared with nonstressed germplasms, respectively, and these data demonstrated that combined cold and drought stress can promote the accumulation of Pro and SP in the leaves of *Poa annua* seedlings, which can increase the resilience of *Poa annua*. In addition, the SS content of the ‘HZ’ germplasm increased the most under the combined cold and drought stress, while the SS content of the ‘XH’ germplasm increased less.

### PCA of physiological and photosynthetic characteristics of *Poa annua* germplasms under different stresses

**Figure 10:**
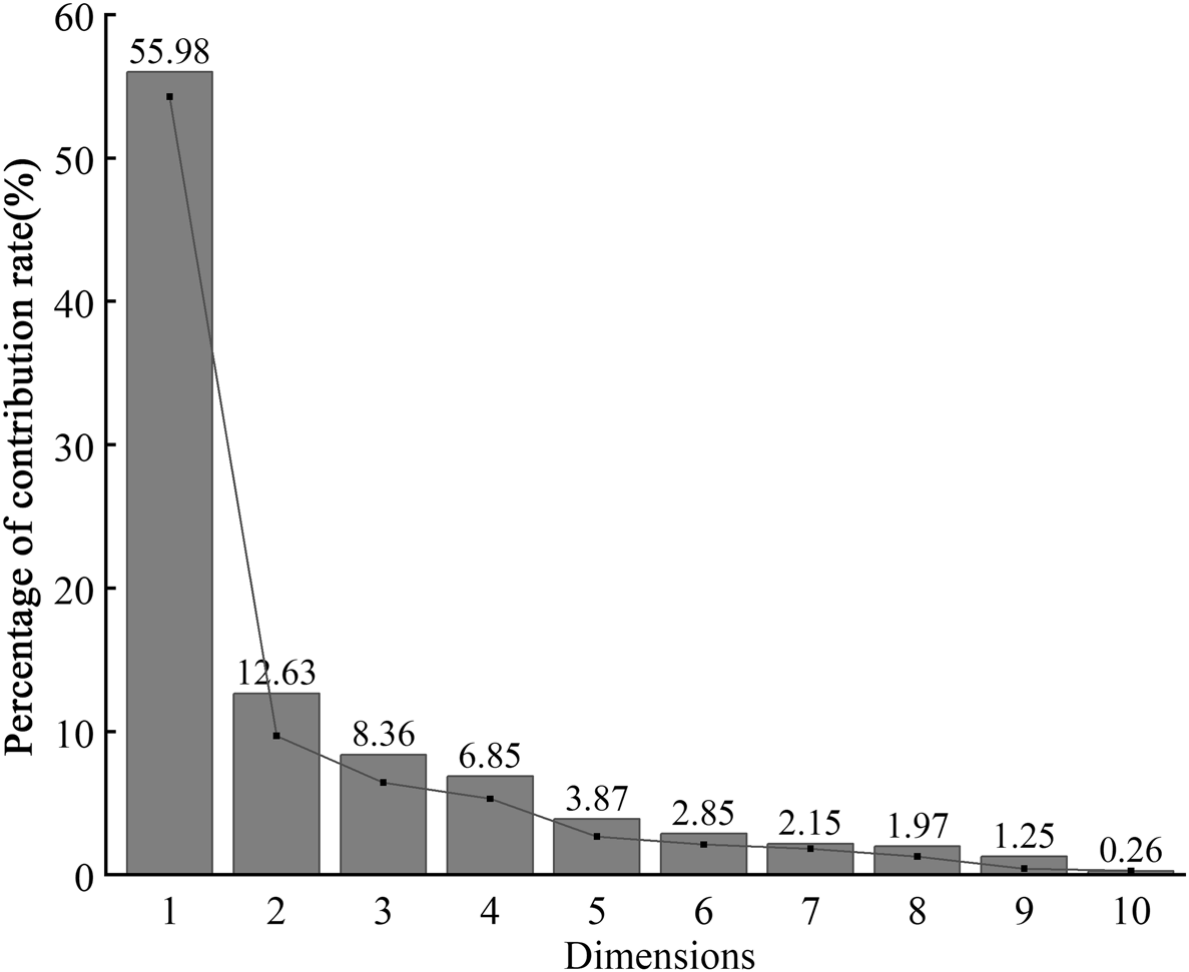
The analysis of the contribution rate of the principal components.

To eliminate differences caused by the basic traits of germplasms from different provenances, relative values were used to describe the responses of plants to combined cold and drought. 30 indexes on leaf functional traits, photosynthetic and physiological of 16 germplasms grown with regular watering (the soil moisture content was 80% of the maximum field capacity) at room temperature (25 °C) and under combined drought (the soil moisture content was 30% of the maximum field capacity) and cold (0 °C) stress were transformed into different principal components. The contribution rates of the variance of the first four principal components of the *Poa annua* seedlings related to stress treatments were 55.98%, 12.63%, 8.36%, and 6.85%, respectively, with a cumulative contribution rate of 83.82%, which represented 83.82% of the comprehensive information in the related indices (Fig. 10). Therefore, only the first two principal components, which could comprehensively reflect the main information in the raw data for each test index, and were selected as effective principal components for analysis.

**Figure 11:**
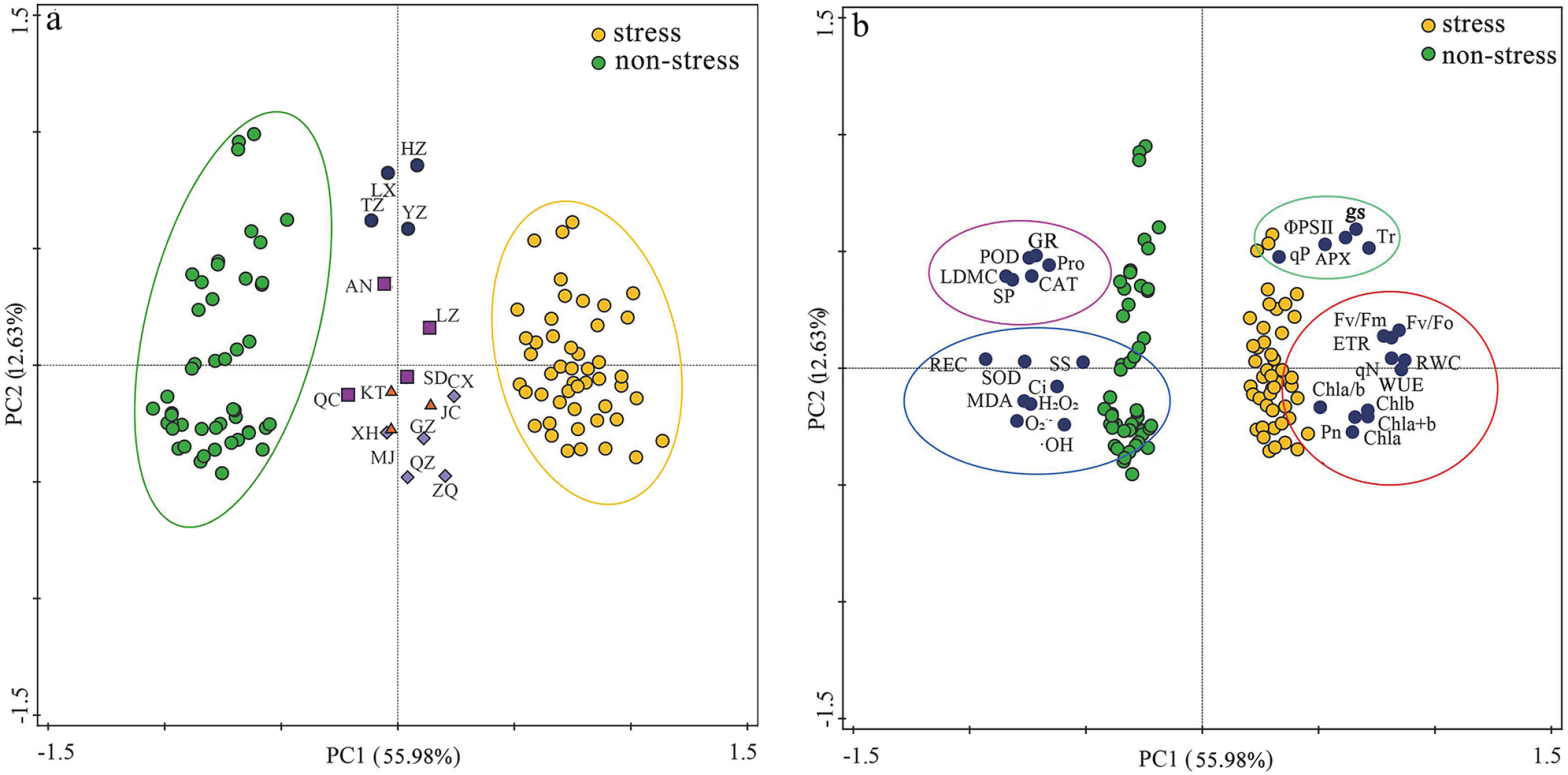
Observation plot showing separation of different treatments and *Poa annua* germplasms in different quadrants (**a**) PCA biplot showing the correlations between leaf functional traits, photosynthetic, and physiological and biochemical characteristics of *Poa annua* germplasms under different combined water and temperature stress (**b**). The abbreviations of the corresponding indicators are shown in Table S1 of additional file 1.

30 indicators were divided into the different treatments and germplasms into four quadrants (Q) in the PCA. Figure 11a reflected a clear separation of the two treatments, with the stressed group located on the right side of PC1 (Q_3_ and Q_4_) and the control group located on the left side of PC1 (Q_1_ and Q_3_). In addition, we found significant segregation among the 16 *Poa annua* germplasms: the ‘LX’, ‘TZ’, and ‘AN’ germplasms were in Q_1_; the ‘QC’, ‘KT’, ‘XH’ and ‘MJ’ germplasms were in Q_2_; the ‘HZ’, ‘YZ’, and ‘LZ’ germplasms were in Q_3_; and the ‘SD’, ‘CX’, ‘JC’, ‘GZ’, ‘QZ’, and ‘ZQ’ germplasms were in Q_4_ (Fig. 11a). The double-label plot showed a strong positive correlation between chlorophyll fluorescence parameters and chlorophyll contents in *Poa annua*. However, in the opposite region, the plot exhibited physiological and biochemical characteristics, indicating that combined cold and drought stress enhanced osmotic regulation capacity, ROS accumulation, and antioxidant enzyme activity (Fig. 11b). Moreover, 30 indexes of *Poa annua* germplasms under different stress conditions were analyzed by PCA, four naturally separated populations were formed. The first group was mainly composed of osmoregulatory substances and antioxidant enzyme activities, namely soluble protein (SP), proline (Pro), peroxidase activity (POD), catalase activity (CAT) and glutathione reductase activity (GR). The second group was mainly characterized by lipid peroxidation and reactive oxygen species levels, which included relative conductivity (REC), malondialdehyde (MDA), superoxide anion (O_2_^·-^), hydrogen peroxide (H_2_O_2_) and hydroxyl radical (·OH). Photosynthetic gas parameters, such as stomatal conductance (gs) and transpiration rate (Tr), mainly characterized the third group. The fourth group was mainly characterized by chlorophyll fluorescence parameters and chlorophyll, including the contents of chlorophyll a (Chl a), chlorophyll b (Chl b), chlorophyll a + b (Chl a + b), chlorophyll a/b (Chl a/b), maximum photochemical efficiency of PSII (Fv/Fm), potential photochemical efficiency of PSII (Fv/Fo), electron translate ratio (ETR), and non-photochemical quenching coefficient (qN).

### Comprehensive evaluation and cluster analysis of *Poa annua* germplasm stress resistance

**Figure 12:**
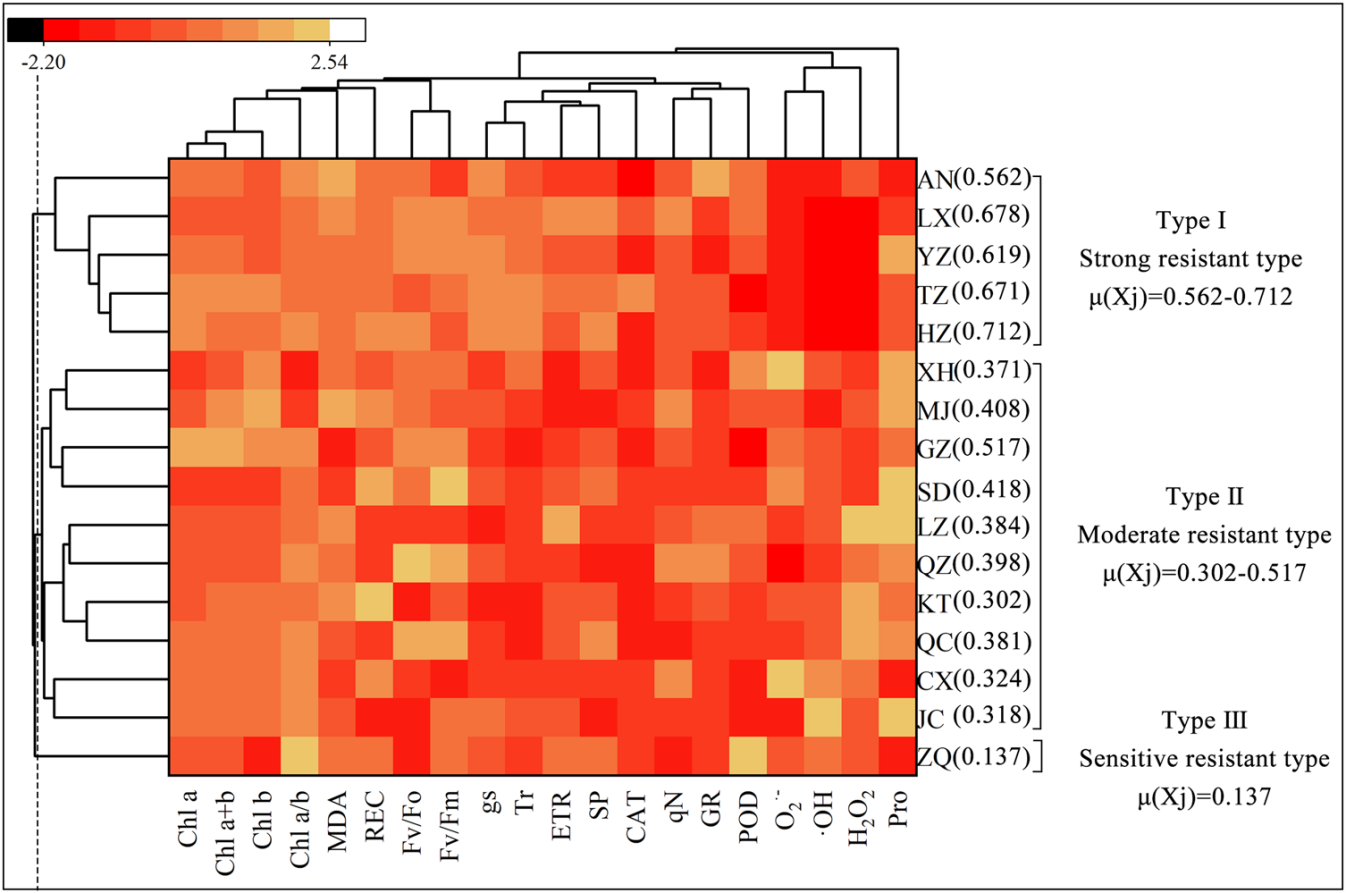
Heat map of cluster analysis for stress resistance among 16 *Poa annua* germplasms.

Plant stress resistance is a quantitative trait controlled by multiple factors. When multiple indexes are used to increase the amount of data processing, the information of each index also crosses and overlaps. However, to eliminate the influence of factors at different orders of magnitude on evaluation results, the PCA method was adopted, and the relative values of each trait were standardized using the affiliation function method based on the PCA screening results. Cluster analysis was performed for 16 germplasms according to the average membership function values (Fig. 12). All germplasms were divided into three categories, cluster I contained five germplasms with strong combined cold and drought resistance, namely, the ‘HZ,’ ‘LX,’ ‘TZ,’ ‘YZ’ and ‘AN’ germplasms. Cluster II contained ten germplasms with moderate combined cold and drought resistance. In cluster III, only the ‘ZQ’ germplasm was more sensitive to combined cold and drought stress.

## Discussion

Temperature and water are the main abiotic stresses limiting plant growth and distribution^25,26^, especially in the arid and semiarid regions. The change of plant external morphology can directly reflect the effect of stress on plants, therefore, it can be used as an index of plant stress resistance^27,28^. In this study, by observing the external morphology of 16 *Poa annua* germplasms that were subjected to combined cold and drought stress for 24 h, we found that the ‘HZ’ germplasm showed less severe damage symptoms than the other germplasms, and the ‘ZQ’ germplasm showed the most severe damage symptoms. The richer the variation in plant traits is, the more the plants can improve their adaptability to different environments^29,30^. Our study found that under combined cold and drought stress, the coefficient of variation of *Poa annua* varied from 8.26% to 74.97%, with an average coefficient of variation of 32.09%, which was much greater than 10%^31^. This indicated that leaf traits showed abundant variation, which was conducive to the screening of combined cold and drought-resistant materials, and provided valuable resources for the breeding of cold-season turfgrass in the future. In contrast, changes in biomass are an integrated expression of plant response to adversity and a visual indicator of plant resilience^27^. In this study, the LDMC of 16 germplasms increased significantly under stress conditions, among them, the ‘ZQ’ germplasm showed the highest increase in LDMC, and the ‘HZ’ germplasm exhibited the lowest increase, which indicated that different germplasms had different growth strategies in response to stress. It was preliminarily speculated that the ‘HZ’ germplasm was more suitable as planting material for lawns in the cold- and drought-prone areas. Cold and drought stress is a special kind of water stress^32,33^. In this study, all 16 *Poa annua* germplasms reflected obvious dehydration symptoms and a dramatic decrease in RWC under combined cold and drought stress, at which time the plants mainly resisted the effects of combined cold and drought stress by increasing their concentration of cytosol.

Photosynthetic pigments are the most basic substances in plants, and changes in their content can, to some extent, reflect the strength of plant stress tolerance^34–36^. In this study, we found that the Chl a, Chl b, Chl a + b contents, and Chl a/b of the 16 germplasms were significantly inhibited after they were subjected to combined cold and drought stress, a phenomenon that suggested that chloroplasts were damaged and chlorophyll was decomposed to varying degrees when plants suffered from combined stress. In addition, the Chl a, Chl b and Chl a + b contents of the ‘TZ’ germplasm were significantly higher than those of the other germplasms after the plants were subjected to combined cold and drought stress, indicating that the ‘TZ’ germplasm has a strong capacity for light energy conversion, absorption and transfer, which allowed it to maintain a high rate of photosynthesis. Under combined stress, the Chl a/b of the ‘XH’ germplasm decreased the most, indicating that combined stress caused damage to the membrane structure of the cysts of the ‘XH’ germplasm and affected the stability of stacking, resulting in significantly weaker resistance than that of the high-resistance germplasm. Gas exchange parameters and chlorophyll fluorescence parameters were adopted as a basis for determining the strength of photosynthesis^37^, and changes in the external environment lead to significant fluctuations in these parameters. Amin et al.^38^ found that the Pn, Tr and gs of cucumber decreased noticeably, while Ci increased with increasing stress levels under combined stress. In this study, the Pn, gs, Tr, and WUE of 16 *Poan annua* germplasms were significantly inhibited under combined cold and drought stress, which was consistent with previous research results^39^. Moreover, the Pn, gs, Tr and WUE of the ‘HZ’ germplasm decreased the least under stress, indicating that the ‘HZ’ germplasm reduced Tr and WUE by self-regulation and reducing gs, and maintaining the relative stability of the microenvironment, thereby maintaining stronger photosynthetic capacity under stress and prolonging the green period of the lawn. Meanwhile, this study demonstrated that under the same stress conditions, the changes in the gas exchange parameters of different germplasms were not consistent. We believe that combined cold and drought stress can affect different plants in different ways and affect photosynthesis processes. Interestingly, Ci sharply increased in the 16 germplasms under combined cold and drought stress, and it can be assumed that the photosynthetic system of *Poan annua* seedlings was impaired due to limitations associated with nonstomatal factors^34^. This may be mainly attributed to the damage of chloroplast structure stability caused by stress, resulting in the damage of leaf photosynthetic apparatus, the reduction of photosynthetic activity, leading to an inhibition of CO_2_ fixation and conversion, and then the increase of Ci. The Fv/Fm and Fv/Fo are important parameters for photochemical reactions, they are commonly used to measure PSII light energy conversion efficiency and possible photoprotective mechanisms in plant leaves. Chlorophyll absorbs and dissipates light energy mainly through chlorophyll fluorescence, electron transfer, and heat dissipation pathways^40^. Plant stress can lead to temporary photoinhibition of photosynthetic organs or direct destruction by adversity, resulting in a decrease in chlorophyll fluorescence parameters^41,42^. The findings of this study demonstrated that the Fv/Fo, Fv/Fm and ΦPSII of the 16 *Poa annua* germplasms were rapidly decreased under combined stress. This implied that the light energy utilization capacity of plants was weakened under combined stress, which hindered the efficiency of electron transfer. Besides, in the process of photosynthesis, electron transfer in plants was coupled with photosynthetic phosphorylation to generate energy-bearing ATP for the synthesis of organic matter by carbon assimilation. Furthermore, noncyclic photosynthetic phosphorylation of NADP accepts Z chain electrons to form NADPH. The inhibition of photosynthetic electron transfer under combined stress inhibits the formation of ATP and NADPH for photosynthetic carbon assimilation, resulting in a decline in the photosynthetic rate of cool-season turfgrass, affecting the normal photosynthesis of turfgrass. In this study, we also found that the Fv/Fo, Fv/Fm and ΦPSII of the ‘ZQ’ germplasm decreased the most under stress, and the Fv/Fo and Fv/Fm had a strong positive correlation, indicating that the changes in Fv/Fm and the potential activity of photoconversion were correlated and synchronized^43,44^. Under the same stress conditions, the ‘ZQ’ germplasm, which was grown in low-altitude, high-temperature, and rainy zones, showed a lower effective photoconversion efficiency, resulting in more inhibition of photochemical reaction. qP, qN, and ETR are important parameters for photosynthetic electron transfer and light energy conversion. In this study, the qP, qN and ETR of the 16 *Poa annua* germplasms were significantly reduced under combined stress. Among which, the qN and ETR of the ‘ZQ’ germplasm had the largest decreases, which indicated that the PSII reaction center of the ‘ZQ’ germplasm had a smaller degree of "opening," i.e., the electron transfer activity of its PSII was lower, and its ability to convert light energy into chemical energy was weaker. This indicated that the photosynthetic electron transfer capacity of the ‘ZQ’ germplasm was weaker, which was not conducive to the production of NADPH and ATP for photosynthetic carbon assimilation, and was even less conducive to the formation of plant photosynthetic products. The qP of the ‘HZ’ germplasm in this study reflected the smallest decrease, further indicating that its leaves absorbed less light energy through thermal dissipation, had a stronger photochemical burst, had a greater ability to use captured light energy, and had a stronger capacity for photosynthesis. The ‘HZ’ germplasm had the smallest decrease in the ETR, illustrating that the ‘HZ’ germplasm could efficiently yield high-energy electrons from the excitation of PSII, and promote rapid photosynthesis.

Notably, the production of ROS in plants is exposed to combined cold and drought stress severely disrupts the dynamic balance between ROS production and removal in plant cells, leading to a series of cell signaling events, such as cellular senescence and apoptosis, which in turn inhibit plant growth and development^45,46^. In this study, the 16 *Poan annua* germplasms accumulated large amounts of O_2_^·-^, H_2_O_2_, and ·OH when subjected to combined stress, which was consistent with previous findings^47^. In this study, we also found that the increase in the O_2_^·-^, H_2_O_2_ and ·OH and other active oxygen of the ‘HZ’ germplasm were the smallest under stress conditions, revealing that the accumulation of ROS was relatively slow and the degree of lipid peroxidation in the cellular membrane was lower in the ‘HZ’ germplasm, which indicated that the ‘HZ’ germplasm had stronger stress resistance. The initial and critical part of plant damage caused by the excessive accumulation of ROS occurs in the cellular membrane. This process can destroy the structural and functional integrity of the membrane. In addition, excessive ROS accumulation causes oxidative damage to cell membrane lipids and tissue senescence, producing toxic oxidation products such as the polyunsaturated fatty acid oxidation product MDA^48^. MDA is a marker of lipid peroxidation associated with oxidative stress and redox signaling, and accumulated MDA could predispose oxidative damage to the plant cell membrane system, especially when stress negatively affects plants^49^. In this study, combined stress increased the MDA contents of the 16 germplasms, and the MDA content of the ‘ZQ’ germplasm increased noticeably compared with those of other germplasms, which demonstrated that cell membrane lipid peroxidation damage and severe damage to cell membrane structure and function occurred in the ‘ZQ’ germplasm. Cell membranes, as semipermeable membranes, play an important role in the exchange and utilization of substances between the interior and exterior of cells, and REC can be used to assess the extent of plant damage occurring^50,51^. Our study reflected that the REC of the 16 *Poa annua* germplasms increased significantly after being subjected to combined cold and drought stress, with the ‘HZ’ germplasm having the smallest REC increase and the ‘ZQ’ germplasm having the largest increase, suggesting that combined cold and drought stress resulted in damage to the cell membrane system of the 16 germplasms and that the ‘ZQ’ germplasm had weak combined cold and drought resistance. Plants under stress-inducing conditions have their protective responses, and the antioxidant enzyme system is an important stress-alleviation strategy. SOD, as the first line of defense in the antioxidant system, can catalyze the disproportionation of O_2_^·-^ to produce O_2_ and H_2_O_2_, and plays a detoxifying role by scavenging O_2_^·-^. For the scavenging of H_2_O_2_ reactive oxygen species, CAT is an enzyme that exclusively scavenges H_2_O_2_, and POD is also involved in the scavenging of reactive oxygen species. APX and GR also indirectly scavenge H_2_O_2_ in chloroplasts. The scavenging of O_2_^·-^ and H_2_O_2_ by the synergistic action of the above enzymes inhibits the formation of highly reactive ·OH and limits the initiation of reactions involving these free radicals for the peroxidation of membrane lipids to reduce plant membrane system injury^52^. Hence, the antioxidant system is considered to be a good indicator of plant tolerance to stress. In this study, we found that the activities of SOD, POD, CAT, APX, and GR were significantly elevated in the 16 *Poa annua* germplasms under combined cold and drought stress, which is consistent with the results of previous studies^53,54^; from this, it can be inferred that plants activated a stress response mechanism to initiate antioxidant enzyme activities during adverse conditions through stress-induced membrane lipid peroxidation and then adapted to the stress and re-established a balance between ROS generation and scavenging^55^. However, Wang and Wei^56^ showed that the CAT activity of *Sorghum bicolor* cv. Dochna seedling leaves were significantly reduced under combined drought and salt stress. During their evaluation of the cold tolerance of different sugarcane varieties, Quan Yiji et al.^57^ found that the activity of POD was significantly reduced under cold stress, which differed from the results of the present study, and it was hypothesized that this difference might be related to the species, the type of stress, and the stage at which the stress was applied. Moreover, the present study was only conducted under indoor simulated conditions, which differed from conditions in the field to a certain extent in terms of environmental factors. Therefore, it is necessary to further explore its precise regulatory mechanism, and the study of a single factor cannot explain the comprehensive regulatory mechanisms of the plants. It is well known that Pro, SS and SP are important osmoregulatory substances in plants. Under stress, the osmoregulatory substances enhance the stability of cell membranes, playing an important role when plants are subjected to stress, and their content can be used as an indicator of plant resistance to stress. It is generally believed that the contents of Pro, SS, and SP are positively correlated with plant resistance^58^. The results of this study showed that the Pro, SS, and SP contents of the 16 *Poa annua* germplasms increased to different degrees under combined cold and drought stress, suggesting that plants reduce their cellular osmotic potential by regulating their Pro, SS and SP contents under stress conditions to alleviate damage from combined cold and drought stress on *Poa annua* seedlings. We also found that the Pro and SP contents of the ‘ZQ’ germplasm and the SS content of the ‘XH’ germplasm increased the least under stress conditions, while the increase in the ‘HZ’ germplasm was the greatest. The osmoregulatory ability of different *Poa annua* germplasms to cope with combined cold and drought stress varies significantly. The stronger resistant plants can actively accumulate proline and regulate the hydrolysis and transformation of soluble sugar, to reduce the cell expansion pressure and membrane damage caused by the decrease of osmotic potential, and thus protect the normal metabolic processes of plants. In addition, the variability in the osmoregulation ability of different germplasms to the same stress may be related to the genetic background (soil, rainfall, temperature, etc.) of the different germplasms, and favorable genes can be identified through the analysis of kinship relationships between seed sources at a later stage of research to provide a theoretical basis for the conservation of favorable genotypes and the selection of germplasms for breeding.

**Figure 13:**
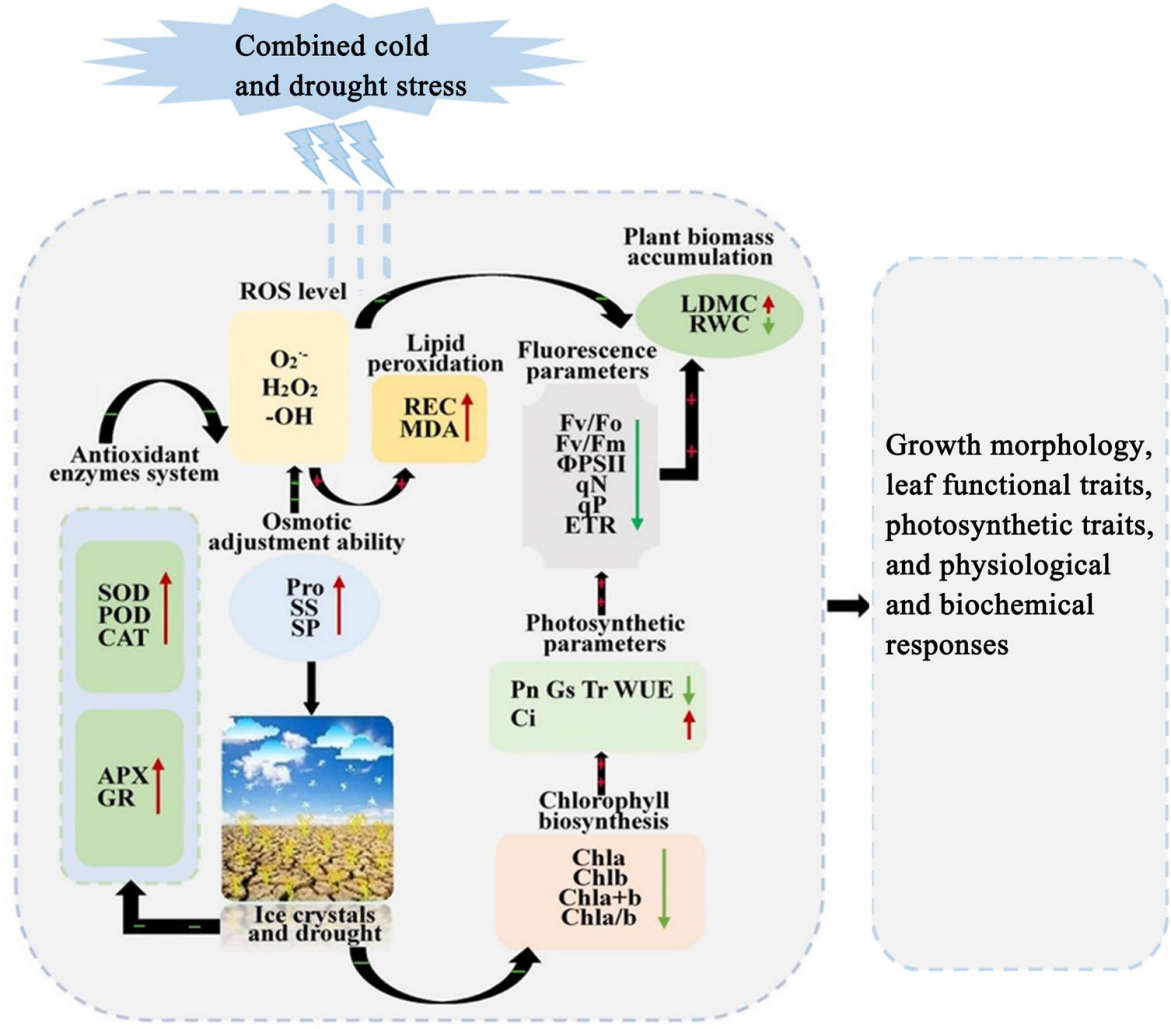
Response mechanism underlying resistance to combined cold and drought stress in *Poa annua* seedlings. The red and green arrows indicated that the corresponding traits were increased and decreased under stress treatment, respectively. " +  + " and " −  − " indicated that the corresponding pathways acted positively and negatively, respectively.

Based on previous research findings^14,44,50,59,60^ and using PCA in this study, we found that the onset and accumulation of stress injury in *Poa annua* seedlings involved different physiological processes. In addition, the effect of stress on plants was alleviated through various mechanisms within cells. For this purpose, we constructed a map of the response mechanisms for combined cold and drought resistance in *Poa annua* (Fig. 13). Briefly, *Poa annua* seedlings activated their antioxidant system and multiple metabolic pathways under combined cold and drought stress, which altered a variety of physiological processes, such as leaf functional traits, chlorophyll biosynthesis, photosynthetic properties, chlorophyll fluorescence parameters, lipid peroxidation levels, osmoregulatory capacity, ROS levels, and antioxidant enzyme systems. Moreover, these physiological responses interacted with each other to further activate secondary and tertiary regulatory networks to prevent or minimize the adverse effects of stress.

## Conclusions

By measuring the external morphology, leaf functional traits, photosynthesis and physiological and biochemical indexes of 16 *Poa annua* germplasms during the seedling stage in response to cold and drought stress, it was found that combined cold and drought stress significantly inhibited photosynthetic pigment content, gas exchange parameters and chlorophyll fluorescence parameters of resistance-sensitive germplasm, which ultimately led to the decline of photosynthesis. However, a series of photosynthesis and physiological activities of strong resistance germplasm were less jeopardized by cold and drought stress, so that photosynthesis could be maintained in a stronger state, and thus showed good resistance to stress. The structure and function of the cell membrane of the resistance-sensitive germplasm were severely impaired, which eventually led to the loss of selective permeability and the extravasation of intracellular electrolytes. The combined cold and drought stress significantly increased the MDA content, O_2_^·-^ , H_2_O_2_ and ·OH, and other reactive oxygen species in *Poa annua* seedlings, and the plants reduced the adverse effects of adversity stress by activating the self-stress mechanisms to increase the activities of SOD, POD, CAT, APX, and GR, and by increasing the osmoregulatory substances such as Pro, SS, and SP. These findings provide potentially valuable resources and information for exploring combined cold and drought-resistance genes in cool-season turfgrasses for breeding.

## Materials and methods

### Plant and culture

Seeds of *Poa annua* from 16 provenances were obtained from May to July 2021 in Gansu and Qinghai Provinces, China, and named after the provenances. We have permission to collect *Poa annua*. The voucher specimen, ‘YZ’, PE 01850519, was identified by Guoliang Zhang and its sheet was deposited in the herbarium PE (https://sweetgum.nybg.org/science/ih/herbarium-list). ‘AN’, PE 00925127, was identified by Guoliang Zhang and its sheet was deposited in the herbarium PE (https://sweetgum.nybg.org/science/ih/herbarium-list). ‘LX’, KUN 1403520, was identified by Wei Qi and its sheet was deposited in the herbarium KUN (https://www.cvh.ac.cn/spms/detail.php). ‘SD’, QYTC QYTC0005973, was identified by Shirong Ma and its sheet was deposited in the herbarium QYTC (https://www.cvh.ac.cn/spms/detail.php). ‘HZ’, IBSC 0125425, was identified by Shaoqing Chen and its sheet was deposited in the herbarium IBSC (https://www.cvh.ac.cn/spms/detail.php). ‘XH’, HNWP 0285609, was identified by Kun Liu and its sheet was deposited in the herbarium HNWP (https://www.cvh.ac.cn/spms/detail.php). Simultaneously, The voucher specimen of ‘MJ’ and ‘QZ’, PE 00925122, was identified by Marina Olonova and its sheet was deposited in the herbarium PE (https://sweetgum.nybg.org/science/ih/herbarium-list). The voucher specimen of ‘CX’, ‘JC’ and ‘KT’, BNU 0020964, was identified by Yi He and its sheet was deposited in the herbarium BUN (https://www.cvh.ac.cn/spms/detail.php). The voucher specimen of ‘TZ’, ‘GZ’ and ‘LZ’, PE 02078373, was identified by Shuliang Hu and Xuezhong Liu and its sheet was deposited in the herbarium PE (https://www.cvh.ac.cn/spms/detail.php). They can be searched in the Chinese Virtual Herbarium (https://www.cvh.ac.cn/index.php). The geographical locations and climatic conditions of provenances are shown in Additional file 1: Table S2. The test was conducted at Gansu Agricultural University, Gansu Province, China (36°5′N, 103°34′E), with an average annual temperature of 10.3 °C and average yearly precipitation of 350 mm. The seeds were sown in plastic pots (18 cm inner diameter, 12 cm height, with holes in the bottom) in June 2022. The cultivated substrate was farmland soil, sand, sheep manure, and organic nutrient soil (7:1:1:1; v/v). Each pot was filled with 1.8 kg of mixed substrate, with a sowing rate of 8 g·m^−2^, and cultivated under natural conditions. Furthermore, the position of the pots was moved periodically to reduce differences in the microenvironment.

### Stress treatment

Two–month–old seedlings were exposed to combined cold and drought stress. The experiment was conducted with a completely randomized design. Seedlings of *Poa annua* from various provenances were used to establish a control group (regular watering at room temperature) and a stress group (combined cold and drought stress), with 3 replicates of each treatment and a total of 96 pots. One week before the initiation of the stress experiment, both groups of plants were weighed and watered daily to maintain the same soil moisture content (the soil moisture content was 80% of the maximum field capacity). After that, the control group plants continued regular watering (the soil moisture content was 80% of the maximum field capacity) at room temperature (25 °C), whereas the plants in the stress group were subjected to natural drought to gradually reduce the soil moisture content to 30% of the maximum field capacity, and then the seedlings were subjected to cold stress, while the soil moisture content remained unchanged. First, seedlings were precooled in an incubator at 15 °C for 24 h. Then, the temperature was reduced to 0 °C at a rate of 5 °C /2 h, followed by combined cold and drought stress for 24 h. A stratified soil coring procedure was used to determine the maximum field capacity of the soil, and the soil water content was determined by a weighing method^61^. After the stress treatment, the seedlings in each group were randomly selected for the determination of growth indices, functional leaves in the upper leaves of seedlings were selected to measure photosynthetic gas exchange parameters and chlorophyll fluorescence parameters, and the remaining leaves were used to determine physiological and biochemical indices.

## Measurement items and methods

### Leaf functional traits

Before the stress treatment, nine seedlings (three per pot) were randomly selected from each group, weighed for fresh weight (FW) and saturated the fresh weight (SFW), then placed in an oven (DHG–9030, Shanghai Yiheng Scientific Instrument Co., Ltd., Shanghai, China) at 75 ◦C to dry until constant weight, and weighed for the dry weight (DW), Finally, leaf dry matter content (LDMC) and relative leaf water content (RWC) were calculated, the morphological changes of leaves before and after stress were photographed and recorded.

### Chlorophyll content

The leaf pigments were extracted using 95% (v/v) ethanol by the method of Zhu et al.^62^. The chlorophyll a (Chl a) and chlorophyll b (Chl b) contents of the extracts were measured at an absorbance of 665 nm and 649 nm by UV–Vis spectrophotometer (Cary 60 UV–Vis, Agilent Technologies Inc., Santa Clara, CA, USA), and the Chl a + b content and Chl a/b were calculated.

### Photosynthetic gas exchange parameters

The photosynthetic parameters were measured in September 2022 on leaves of *Poa annua* seedlings in the non-stress and stress groups. Functional leaves with similar growth conditions at the upper edge of the plant were selected for measurement of the net photosynthetic rate (Pn), intercellular CO_2_ concentration (Ci), stomatal conductance (gs), and transpiration rate (Tr) using a portable photosynthetic instrument (Ciras-2, MA01913) every morning (9:00–11:00 on a sunny day). Besides, Water use efficiency (WUE) was expressed as WUE = Pn/Tr.

### Chlorophyll fluorescence parameters

Chlorophyll fluorescence parameters were measured using a portable chlorophyll fluorometer IMAGING-PAM (WALZ, Nuremberg, Germany) from 9:00 to 11:00.

### REC and MDA content

Relative conductivity (REC) was determined using the electrical conductivity method^63^. 0.1 g of fresh leaves were placed in a test tube containing 10 mL of ultrapure water and then incubated at 40 °C for 0.5 h to determine the conductivity of the solution (R_1_), followed by heating in boiling water for 15 min to measure the conductivity of the solution (R_2_). REC was calculated as REC (%) = R_1_/R_2_*100%.

A thiobarbituric acid (TBA) colorimetric method was utilized to determine malondialdehyde (MDA) content^64^. Briefly, 0.1 g of leaves were homogenized in trichloroacetic acid (5%) extract and then centrifuged at 10,000 *rpm*/min for 10 min at 4 °C. Then, 0.1 mL of supernatant was mixed thoroughly with 0.1 mL TBA, and boiled in a water bath (HWS–26, Shanghai Yiheng Scientific Instrument Co., Ltd., Shanghai, China) for 30 min, and after cooling, the solutions were centrifuged at 12,000 *rpm*/min for 10 min, and absorbance at 532 nm and 600 nm was recorded by UV–Vis spectrophotometer.

### Reactive oxygen levels

The superoxide anion radical (O_2_^·-^) was determined by the hydroxylamine oxidation method^65^. Briefly, 0.1 g leaves were homogenized in 1 mL of phosphate buffer (50 mM, pH 7.8), and then centrifuged at 12,000 rpm/min for 20 min at 4 °C. 0.5 mL of supernatant was mixed thoroughly with 0.5 mL of phosphate buffer and 1 mL of hydroxylamine hydrochloride solution, and then the mixture was kept in a water bath at 25 °C for 1 h. The absorbance was measured with a UV–Vis spectrophotometer at 530 nm.

The H_2_O_2_ content was employed by the acetone method^66^. Firstly, 0.15 g leaves were homogenized in 1.5 mL of acetone and then centrifuged at 1000 rpm/min for 10 min at 4 °C. The 1 mL supernatant was mixed thoroughly with 0.1 mL of Sulfuric acid (5%) and 0.2 mL of NH_3_·H_2_0. When a precipitate was formed, centrifuge at 3000 *rpm*/min for 10 min and discard the supernatant. Then 5 mL of H_2_SO_4_ (2 M) was added to the precipitate, and the absorbance was measured at 415 nm by UV–Vis spectrophotometer after the precipitate was completely dissolved.

The scavenging rate of hydroxyl radicals (·OH) was determined by the salicylic acid method^67^. 0.1 g of leaves were homogenized in 1 mL of distilled water and then centrifuged at 4 °C at 10,000 *rpm*/min for 10 min. Then, 0.5 mL of the supernatant was added to 1 mL FeSO_4_ (9 mM), 1 mL ethanol-salicylic acid (9 mM), the appropriate amount of deionized water and 1 mL H_2_O_2_ (8.8 mM), and then kept at 37 °C for 15 min. Finally, the absorbance at 510 nm was measured by UV–Vis spectrophotometer.

### Antioxidant enzyme activity

The antioxidant enzymes were measured by the method of Khalid et al.^68^ with some improvements. 0.2 g of leaves were homogenized in 5 mL of pre-cooled phosphate buffer (50 mM, pH 7.8) and then centrifuged at 5000 rpm/min for 10 min at 4 °C, and the resulting supernatant was the crude enzyme extract. The activities of superoxide dismutase (SOD), peroxidase (POD), catalase (CAT), ascorbate peroxidase (APX), and glutathione reductase (GR) were determined.

The activity of SOD was determined by the nitro-blue tetrazolium color development method. 1.5 mL phosphate buffer (0.05 M), 0.3 mL methionine solution (130 mM), 0.3 mL nitro-blue tetrazolium solution (750 µM), 0.3 mL disodium ethylenediaminetetraacetic acid solution (100 µM), 0.3 mL vitamin B_2_ (20 µM), 0.1 mL enzyme solution, and 0.5 mL distilled water were added to the tubes, and the tubes were exposed to 4000 Lux illumination for 20 min at 25 °C. After the reaction, the absorbance at 560 nm was measured by UV–Vis spectrophotometer immediately.

The activity of POD was detected by the guaiacol method. Briefly, 28 µL guaiacol was added to 50 mL phosphate buffer (0.2 M, pH 6), heated and stirred to dissolve completely. After cooling, 19 µL H_2_O_2_ (30%) was added to the phosphate solution. Then 3 mL of the above reaction solution was mixed with 0.1 mL of the enzyme solution and the change in absorbance at 470 nm within 40 s was measured by UV–Vis spectrophotometer.

The activity of CAT was determined by UV spectrophotometry. Specifically, 0.155 mL H_2_O_2_ (30%) was dropped into 100 mL phosphate buffer (0.15 M, pH 7.0) and shaken well. Then 2.9 mL of the reaction solution was mixed with 0.1 mL of the enzyme solution, and finally, the change in absorbance at 240 nm within 40 s was measured by UV–Vis spectrophotometer.

The activity of APX was detected by the ascorbic acid method. Specifically, 0.1 mL of enzyme solution was added to phosphate buffer (0.05 M, pH 7.0), followed by 0.15 mL of ascorbic acid (5 mM) and 0.15 mL H_2_O_2_ (20 mM), and the change in absorbance at 290 nm within 40 s was measured immediately by UV–Vis spectrophotometer.

The activity of GR was measured by spectrophotometrically. The 0.3 mL of enzyme solution was dropped into 1.7 mL Hepes, 100 µL NADPH and 100 µL GSSG. Finally, the change in absorbance at 340 nm was measured by UV–Vis spectrophotometer immediately within 40 s.

### Osmoregulatory substances

The content of proline (Pro) was determined using the acid ninhydrin method^69^. Briefly, 0. 1 g leaves were added to 1 mL 5-sulfosalicylic acid dihydrate (3%) and extracted in a boiling water bath for 10 min, then centrifuged at 1000 rpm/min for 10 min at 25 °C. 1 mL of the supernatant was taken and mixed with 1 mL acetic acid and 1 mL acidic ninhydrin. Subsequently, it was heated in a water bath for 30 min, after cooling, 2 mL toluene was added, shaken well, and standing at room temperature for 1 min. The supernatant was taken and absorbance at 520 nm was recorded by UV–Vis spectrophotometer.

The soluble sugar (SS) content was determined by the anthrone colorimetric method^65^. 0.2 g leaves in 10 mL of distilled water, and then extracted by heating in a water bath for 30 min. After cooling, the extract was filtered into a 50 mL volumetric bottle, the test tube and residue were rinsed repeatedly, and set the volume to scale. 1 mL of extract was thoroughly mixed with 1.5 mL of distilled water, 0.5 mL of anthrone ethyl acetate and 5 mL of H_2_SO_4_, immediately kept in boiling water for 1 min, and after cooling, the absorbance at 630 nm was recorded by UV–Vis spectrophotometer.

The soluble protein (SP) content was tested by coomassie brilliant blue method^65^. The 0.2 g leaves were homogenized in 2 mL phosphate buffer (50 mM, pH 7.8), and centrifuged 12,000 rpm/min for 20 min at 4 °C. The 0.1 mL enzyme solution was thoroughly mixed with 2.9 mL coomassie brilliant blue, and left for 2 min. Then the absorbance at 595 nm was recorded by UV–Vis spectrophotometer.

### Statistical analysis

Using the SPSS 20.0 (SPSS, Inc., Chicago, IL, USA), the data were subjected to ANOVA and multiple comparisons. Two-way analyses of variance (ANOVA) were conducted to detect the effects of provenance and combined drought and cold stress and their interactions. All statistical effects were considered significant at *P* < 0.05, and Origin 2021.0 was used for plotting figures, principal component analysis (PCA), and cluster analysis heat maps. The data were comprehensively analyzed using the fuzzy mathematical membership function method to evaluate the combined cold and drought tolerance of the 16 *Poa annua* germplasms. The value of the affiliation function was calculated as follows: *µ*(*Xj*) = (*Xj*−*X*min)/(*X*max−*X*min), and in-verse membership function value: *µ(Xj*) = 1−(*Xj*−*X*min)/(*X*max-*X*min), where *µ*(*Xj*) denotes the value of the affiliation function of the *j*th index; *Xj* denotes the value of the *j*th indicator value; *X*min indicates the *j*th indicator minimum value; *X*max indicates the *j*th indicator maximum value. In addition, the coefficient of genetic variation (CV%) for single traits of *Poa annua* under each treatment was calculated:(CV%) = SD/ X̅, where SD represents the standard deviation, and X̅ represents the mean value of single traits among the germplasm under each treatment.

### Supplementary Information

Supplementary Tables.

## Data Availability

Data available on reasonable request from corresponding author.
