# Supplementary material for: Effects of combined extreme cold and drought stress on growth, photosynthesis, and physiological characteristics of cool-season grasses
Source: Sci Rep. 2024 Jan 2;14:116. doi: 10.1038/s41598-023-49531-1 (PMC10762181; doi:10.1038/s41598-023-49531-1)
Supplement: Supplementary file 1 — Supplementary Tables. [file 41598_2023_49531_MOESM1_ESM.pdf]

Table S1. Main interactions and coefficients of variation of growth and photosynthetic and physiological traits of different *Poa annua* seedlings under the stress treatments.

Trait	Provenance(P)	Temperature(T)	PxT Interaction	CV(CK)(%)	CV(T)(%)
LDMC	F=4.84($p<0.001$)	F=296.39($p<0.001$)	F=3.41($p<0.001$)	12.27%	12.28%
RWC	F=17.12($p<0.001$)	F=1695.90($p<0.001$)	F=9.26($p<0.001$)	13.74%	14.70%
Chla	F=36.04($p<0.001$)	F=596.84($p<0.001$)	F=22.76($p<0.001$)	50.26%	30.48%
Chlb	F=14.62($p<0.001$)	F=430.10($p<0.001$)	F=10.59($p<0.001$)	26.65%	13.74%
Chla/b	F=35.51($p<0.001$)	F=105.08($p<0.001$)	F=24.34($p<0.001$)	32.01%	29.18%
Chla+b	F=15.26($p<0.001$)	F=797.43($p<0.001$)	F=5.01 ($p<0.001$)	37.61%	16.22%
Pn	F=21.10($p<0.001$)	F=2837.23($p<0.001$)	F=28.48 ($p<0.001$)	29.27%	44.36%
Ci	F=32.78($p<0.001$)	F=448.38($p<0.001$)	F=8.58 ($p<0.001$)	15.03%	14.35%
gs	F=85.19($p<0.001$)	F=1249.83($p<0.001$)	F=15.06($p<0.001$)	25.35%	74.97%
Tr	F=47.43($p<0.001$)	F=1016.97($p<0.001$)	F=9.69($p<0.001$)	21.89%	64.48%
WUE	F=20.87 ($p<0.001$)	F=343.73 ($p<0.001$)	F=4.83 ($p<0.001$)	33.92%	46.98%
Fv/Fo	F=21.33($p<0.001$)	F=1820.21($p<0.001$)	F=26.28($p<0.001$)	17.32%	46.52%
Fv/Fm	F=11.10($p<0.001$)	F=875.81($p<0.001$)	F=8.56($p<0.001$)	6.60%	22.82%
ΦPSII	F=31.82($p<0.001$)	F=315.97($p<0.001$)	F=2.89 ($p<0.05$)	35.27%	69.27%
qN	F=4.81 ($p<0.001$)	F=398.03($p<0.001$)	F=2.27($p<0.05$)	5.30%	8.26%
qP	F=164.00($p<0.001$)	F=237.95($p<0.001$)	F=2.19($p<0.05$)	46.91%	64.35%
ETR	F=50.89 ($p<0.001$)	F=1353.86($p<0.001$)	F=7.74($p<0.001$)	24.72%	47.76%
REC	F=4.63($p<0.001$)	F=2729.41($p<0.001$)	F=5.21($p<0.001$)	25.92%	11.34%
MDA	F=51.57($p<0.001$)	F=3332.63($p<0.001$)	F=26.58($p<0.001$)	9.37%	16.54%
$O_2^{\cdot-}$	F=30.78($p<0.001$)	F=1501.05 ($p<0.001$)	F=38.59 ($p<0.001$)	11.66%	30.22%
H_2O_2	F=9.85 ($p<0.001$)	F=18.78 ($p<0.001$)	F=2.45($p<0.05$)	21.70%	19.17%
·OH	F=98.32($p<0.001$)	F=909.95($p<0.001$)	F=8.81($p<0.001$)	28.12%	22.47%
SOD	F=39.76($p<0.001$)	F=39.76($p<0.001$)	F=10.92 ($p<0.001$)	46.38%	22.42%
POD	F=34.60 ($p<0.001$)	F=34.60 ($p<0.001$)	F=22.71($p<0.001$)	31.39%	36.37%
CAT	F=19.23 ($p<0.001$)	F=19.23 ($p<0.001$)	F=7.42($p<0.001$)	43.99%	31.40%
APX	F=30.24($p<0.001$)	F=30.24($p<0.001$)	F=10.52($p<0.001$)	50.54%	40.17%
GR	F=105.80($p<0.001$)	F=105.80($p<0.001$)	F=25.47($p<0.001$)	43.63%	35.21%
Pro	F=90.16($p<0.001$)	F=2010.95($p<0.001$)	F=35.02($p<0.001$)	66.66%	46.63%
SS	F=26.02($p<0.001$)	F=26.02 ($p<0.001$)	F=7.53($p<0.001$)	28.56%	19.71%
SP	F=12.93($p<0.001$)	F=12.93($p<0.001$)	F=7.39($p<0.001$)	6.40%	10.36%

LDMC, leaf dry matter content; RWC, relative leaf water content; Chl a, chlorophyll a content; Chl b, chlorophyll b content; Chl a/b, chlorophyll a:b ratio; Chl a+b, chlorophyll a+b content; Pn, net photosynthetic rate; Ci, intercellular CO_2 concentration; Gs, stomatal conductance; Tr, transpiration rate; WUE, instantaneous water use efficiency; Fv/Fo, potential photochemical efficiency of PSII; Fv/Fm, maximum photochemical efficiency of PSII; ΦPSII, the actual photochemical quantum efficiency of PSII; qN, non-photochemical quenching coefficient; qP, photochemical quenching; ETR, electron transfer ratio; REC, relative electrical conductivity; MDA, malondialdehyde content, $O_2^{\cdot-}$, superoxide anion; H_2O_2, hydrogen peroxide; ·OH, hydroxyl radical; SOD, superoxide dismutase activity; POD, peroxidase activity; CAT, catalase activity; APX, ascorbate peroxidase activity; GR, glutathione reductase activity; Pro, proline content; SS, soluble sugar content; SP, soluble protein content. Same as below.

Table S2. Geography of the main distribution areas of the 16 wild *Poa annua* germplasms for testing

Number	Collection Place	Longitude	Latitude	Elevation /m	Average annual rainfall /mm	Average annual temperature /°C	Habitat
SD	Shandan County, Zhangye City, Gansu Province	101°36′	38°72′	1781	328	8	In front of the house and behind the house
HZ	Huanzhong District, Xining City, Qinghai Province	101°78′	36°51′	2677	560	3	In front of the house and behind the house
JC	Jingchuan County, Pingliang City, Gansu Province	107°37′	33°47′	1193	555	10	In front of the house and behind the house
ZQ	Zhouqu County, Gannan Tibetan Autonomous Prefecture, Gansu Province	104°22′	33°46′	1420	689	12	River valley
QC	Qingcheng County, Qingyang City, Gansu Province	107°60′	35°89′	1316	572	10	In front of the house and behind the house
KT	Kongdong District, Pingliang City, Gansu Province	106°53′	35°59′	1540	477	9	In front of the house and behind the house
LX	Linxia County, Linxia Hui Autonomous Prefecture, Gansu Province	103°08′	35°49′	2012	573	6	Green belt
QZ	Qinzhou District, Tianshui City, Gansu Province	105°44′	34°30′	1350	536	10	In front of the house and behind the house
MJ	Maiji District, Tianshui City, Gansu Province	105°58′	34°21′	1603	560	11	Roadside
AN	Anning District, Lanzhou City, Gansu Province	103°42′	36°52′	1543	325	10	Green belt

YZ	Yuzhong County, Lanzhou City, Gansu Province	104°11′	36°40′	1940	390	7	Green belt
LZ	Liangzhou District, Wuwei City, Gansu Province	102°63′	37°30′	1523	144	9	In front of the house and behind the house
CX	Chongxin County, Pingliang City, Gansu Province	107°02′	35°31′	1149	647	10	In front of the house and behind the house
GZ	Ganzhou District, Zhangye City, Gansu Province	103°15′	16°99′	1478	128	7	Green belt
XH	Xihe County, Longnan City, Gansu Province	104°20′	33°47′	1736	533	8	In front of the house and behind the house
TZ	Tianzhu County, Wuwei City, Gansu Province	101°4′	38°96′	2783	397	4	Green belt
